# STAYING ACTIVE IN DAILY LATER LIFE: BENEFITS ON HYPERTENSION AND DEPRESSION DURING THE COVID-19 PANDEMIC

**DOI:** 10.1093/geroni/igae098.3302

**Published:** 2024-12-31

**Authors:** Jeehoon Kim, Mi-Jeong Lee

**Affiliations:** Idaho State University, Pocatello, Idaho, United States; University of Hawaiʻi at Mānoa, Honolulu, Hawaii, United States

## Abstract

Maintaining active engagement is important to reduce disease risks in later life. Given greater restrictions during the COVID-19 pandemic, adults ages of 71-100 might have limited opportunities to engage in daily exercise. However, less is known whether physical activity is related to cardiometabolic diseases and mental health in 70-plus adults during the pandemic. Using the 2021 National Health and Aging Trends Study (NHATS) accelerometry data (n=740), this study conducted a latent profile analysis based on four activity-related variables including total activity count per minute (TACM). Multinomial logistic regression analysis was performed (base outcome=high level group). Predictors included BMI, incidence of hypertension, type 2 diabetes, cancer, heart diseases, depression and dementia, NHATS summary score of Short Physical Performance Battery, and demographic variables. Three classes were profiled based on the activity level: low (26%, 683 TACM), middle (56%, 1,227 TACM), and high level (18%, 1,829 TACM). Low- and middle-level older adults were more likely than high-level to be overweight and had higher incidence of hypertension and mild depression. Low-level older adults tended to have worse summary scores of physical performance than high-level group. Self-reported walking or vigorous physical activity participation was not associated with physical activity level measured with accelerometer. P-values are less than.05 for all findings. Staying active in daily life was correlated with better health outcomes, lower prevalence of hypertension and mild depression, in 70-plus adults during the COVID-19 pandemic. Intervention strategies to promote physical activity in later life, thus helping reduce risks of cardiometabolic diseases, will be discussed.

